# Enteropathogenic *Escherichia coli* O80:H2 in Young Calves with Diarrhea, Belgium

**DOI:** 10.3201/eid2312.170450

**Published:** 2017-12

**Authors:** Damien Thiry, Marc Saulmont, Shino Takaki, Klara De Rauw, Jean-Noël Duprez, Atsushi Iguchi, Denis Piérard, Jacques G. Mainil

**Affiliations:** University of Liège, Liège, Belgium (D. Thiry, S. Takaki, J.-N. Duprez, J.G. Mainil);; Association Régionale de Santé et d’Identification Animales, Ciney, Belgium (M. Saulmont);; Universitair Ziekenhuis Brussel, Brussels, Belgium (K. De Rauw, D. Piérard);; University of Miyazaki, Miyazaki, Japan (S. Takaki, A. Iguchi)

**Keywords:** diarrheic calves, enteropathogenic *Escherichia coli*, serotype O80:H2, *eae-ξ* gene, bacteria, *Escherichia coli*, enteric infections, zoonoses, *E. coli*, Belgium, farms, EPEC, diarrhea, AE-STEC

## Abstract

Serogroup O80 was detected in 40% of 104 enteropathogenic *Escherichia coli* isolates from calves with diarrhea from 42 farms in Belgium during 2008‒2015. These isolates harbored the *eae-ξ* and *fliC_H2_* genes, similar to the O80 attaching-effacing Shigatoxigenic *E. coli* isolates found in humans in France. This strain might be emerging.

Enteropathogenic and attaching-effacing Shigatoxigenic *Escherichia* *coli* (EPEC and AE-STEC) cause bloody diarrhea in humans and young calves. For clarity, we use the term AE-STEC instead of enterohemorrhagic *E. coli*, similar to a previous publication (*1*), to refer to STEC isolates from animals that produce attaching-effacing lesions. EPEC and AE-STEC that infect humans are diverse and comprise scores of serotypes (*2*); in contrast, most calf AE-STEC strains comprise a few serotypes, mostly O5:H-, O26:H11, O111:H-, and O118:H16 (*3*). The O26:H11 serotype is also the most common among calf EPEC. However, most serotypes that infect calves have not been identified (*3*). Therefore, during November 2008‒June 2015, we conducted a study on 104 EPEC and 153 AE-STEC isolates collected from the feces or the intestinal contents of calves suffering diarrhea (1 isolate/calf) at the Association Régionale de Santé et d’Identification Animales in Ciney, Belgium. Isolates were screened by PCR for genes of the 10 most pathogenic and common calf and human O serogroups: O5, O26, O103, O104, O111, O118, O121, O145, O157, and O165. Confirming published results (*3*), 80% (122/153) of AE-STEC isolates and only 21% (22/104) of EPEC isolates tested positive for 1 of these (J.G. Mainil, unpub. data) (*4*). We sought to further characterize this collection of calf EPEC with unidentified O serogroups.

We submitted 9 calf EPECs with unidentified serogroups to the O-typing multiplex PCR platform (*5*); 6 of 9 EPEC isolates contained the O80 serogroup‒encoding gene, and 3 belonged to 3 other O serogroups. We subsequently performed an O80 serogroup‒specific PCR (*5*) of all 31 AE-STEC and 82 EPEC isolates with unidentified serogroups, along with one O80-positive *E. coli* strain and negative controls; 42 EPEC isolates and the O80-positive *E. coli* strain but no AE-STEC isolates or negative controls tested positive.

We further tested the calf EPEC isolates and 3 human Shiga toxin 2‒encoding gene (*stx2*)‒positive AE-STEC O80 isolates from the STEC National Reference Center (Brussels, Belgium) by PCR for *fliC_H2_* and *eae-ξ* genes found in human AE-STEC O80 strains. For amplifying *eae-ξ*, we used previously published PCR conditions (*6*), and for amplifying *fliC_H2_*, we used primers H2_F (5′-TGATCCGACATCTCCTGATG-3′) and H2_R (5′-CCGTCATCACCAATCAACGC-3′) and the following thermocycler conditions: initial denaturation at 94°C for 1 min; 30 cycles of denaturation for 30 s at 94°C, annealing for 30 s at 58°C, and elongation for 1 min at 72°C; and final elongation at 72°C for 2 min. All 42 calf EPEC and 3 human AE-STEC isolates tested positive by both PCRs.

Among the 104 calf EPEC isolates, O80:H2 was frequently found (40% were PCR positive) and, thus, could be considered emerging. Indeed, the EPEC O80 isolates were isolated from calves from 42 farms. The yearly EPEC O80:H2 isolation rate varied from 12% in 2009 to 40%–50% during 2010‒2013 to as high as 73% for the first 6 months of 2015 (Table). In comparison, the rate for EPEC O26 serotype was 5%‒25%. Although prevalence data before 2009 are lacking, EPEC O80 isolates have been found infrequently in animals: 8 in dead poultry (*7*,*8*), 1 in a piglet with diarrhea (*9*), 1 in a healthy cow (*9*), and 5 in lambs with diarrhea (*10*). However, even fewer AE-STEC O80 isolates have been found: 2 in healthy cattle (*6*); 1 in a calf with diarrhea in January 1987 (J.G. Mainil, unpub. data); and 1 in raw cow’s milk cheese (*9*). According to the literature, the O80:H2 serotype might be emerging in France, where human cases of AE-STEC O80:H2 have been reported (*9*).

**Table:** Comparison of yearly isolation rates of EPEC O80:H2 and O26 from calves with diarrhea, Belgium, January 2009‒June 2015*

Year	EPEC		
	Total no.	O80:H2, %	O26, %
2009	17	24	12
2010	19	47	5
2011	12	50	33
2012	9	33	22
2013	15	47	20
2014	20	25	25
2015	11	73	9
Total	104	41	16

*In 2008, only isolates collected in November and December were studied, and 1 EPEC was identified. EPEC, enteropathogenic *Escherichia coli*.

Molecular virulotyping results indicate that our calf EPEC O80 isolates appeared to be more closely related to human AE-STEC (because they all harbored *eae*-ξ and *fliC_H2_*) than to ovine and poultry EPEC O80 (which usually harbor *eae-*β and *fliC_H26_*) (J.G. Mainil, unpub. data) (*8*,*10*). Further studies are needed to characterize these calf EPEC O80:H2 isolates, and the isolation rate of EPEC O80:H2 in calves with diarrhea must be tracked. Additional PCR virulotyping should be performed with our isolates to identify, if present, other EPEC-related virulence genes and extraintestinal *E. coli*‒related virulence genes. Some genes could be located on plasmids, like those that were found in AE-STEC O80:H2 patients with bacteremia and internal organ infections (*9*), although the infected calves from which our isolates were taken did not show evidence of septicemia before or after necropsy. The relationship among calf EPEC O80:H2 isolates (which are all independent isolates, not constituting a single strain until proven otherwise) and between calf and human isolates needs to be further characterized with pulsed-field gel electrophoresis and whole-genome sequencing. The prevalence of O80:H2 EPEC and AE-STEC in healthy cattle at slaughterhouses and farms in Belgium should be examined. Finally, we need to determine whether these calf EPEC O80:H2 isolates are true EPEC, AE-STEC derivatives that have lost *stx* genes, or AE-STEC precursors that could acquire *stx* genes in the future. This work will aid in the detection, prevention, and control of this potentially emerging pathogen.
